# Exaggerated frontoparietal control over cognitive effort-based decision-making in young women with anorexia nervosa

**DOI:** 10.1038/s41380-024-02712-4

**Published:** 2024-08-28

**Authors:** Joseph A. King, Fabio Bernardoni, Andrew Westbrook, Franziska M. Korb, Ilka Boehm, Arne Doose, Daniel Geisler, Katrin Gramatke, Inger Hellerhoff, Sylvia Wolff, Alexander Strobel, Thomas Goschke, Veit Roessner, Stefan Ehrlich

**Affiliations:** 1 Division of Psychological and Social Medicine and Developmental Neurosciences, Faculty of Medicine, Technische Universität Dresden, Dresden, Germany; 2 Department of Psychiatry, Rutgers University, Piscataway, NJ USA; 3 Chair of General Psychology, Faculty of Psychology, Technische Universität Dresden, Dresden, Germany; 4 Eating Disorder Research and Treatment Center, Dept. of Child and Adolescent Psychiatry, Faculty of Medicine, Technische Universität Dresden, Dresden, Germany; 5 Department. of Psychotherapy and Psychosomatic Medicine, Faculty of Medicine, Technische Universität Dresden, Dresden, Germany; 6 Chair of Differential and Personality Psychology, Faculty of Psychology, Technische Universität Dresden, Dresden, Germany; 7 Department. of Child and Adolescent Psychiatry, Faculty of Medicine, Technische Universität Dresden, Dresden, Germany

**Keywords:** Neuroscience, Psychiatric disorders, Psychology

## Abstract

Effortful tasks are generally experienced as costly, but the value of work varies greatly across individuals and populations. While most mental health conditions are characterized by amotivation and effort avoidance, individuals with anorexia nervosa (AN) persistently engage in effortful behaviors that most people find unrewarding (food restriction, excessive exercise). Current models of AN differentially attribute such extreme weight-control behavior to altered reward responding and exaggerated cognitive control. In a novel test of these theoretical accounts, we employed an established cognitive effort discounting paradigm in combination with fMRI in young acutely underweight female patients with AN (*n* = 48) and age-matched healthy controls (HC; *n* = 48). Contrary to the hypothesis that individuals with AN would experience cognitive effort (operationalized as N-back task performance) as less costly than HC participants, groups did not differ in the subjective value (SV) of discounted rewards or in SV-related activation of brain regions involved in reward valuation. Rather, all group differences in both behavior (superior N-back performance in AN and associated effort ratings) and fMRI activation (increased SV-related frontoparietal activation during decision-making in AN even for easier choices) were more indicative of increased control. These findings suggest that while effort discounting may be relatively intact in AN, effort investment is high both when performing demanding tasks and during effort-based decision-making; highlighting cognitive overcontrol as an important therapeutic target. Future research should establish whether exaggerated control during effort-based decision-making persists after weight-recovery and explore learning the value of effort in AN with tasks involving disorder-relevant effort demands and rewards.

## Introduction

Effort, the goal-directed exertion of physical or mental energy, is typically experienced as resource-depleting, costly and aversive [1–4]. As such, we tend to avoid demanding tasks and prefer less effortful ones unless working harder is “worth it” [5–7]. Paradoxically, this also implies that we value the effort we invest [8]. Indeed, we sometimes engage in effortful tasks for their own sake (e.g. solving puzzles) and often experience the fruit of our labor (e.g. puzzle solved) as sweeter when we work harder [9]. The extent to which people are willing to exert effort for rewards varies greatly across individuals [10–12]. Laboratory measures of effort-based cost–benefit decision-making including “effort discounting” [i.e. the decrease in the subjective value (SV)/increase in subjective cost of a reward as a function of the amount of effort needed to obtain it] reliably distinguish clinical populations characterized by motivational deficits from healthy controls (HC; [13, 14]). In contrast, little is known about effort-based decision-making in people with “too much” motivation, such as individuals with anorexia nervosa (AN); a disorder characterized by excess goal pursuit, perfectionism and overcontrol [15–17]. Here, we employed a cognitive effort discounting paradigm (COGED; [18, 19]) with a demonstrated ability to discriminate between groups known to differ in cognitive motivation [20, 21], in combination with fMRI, to test the hypothesis of reduced discounting (i.e. subjective effort cost) and explore the associated neural correlates of cost–benefit valuation and decision-making in individuals with AN relative to HC.

The enigmatic capacity of people with AN to adhere to a strict (and maladaptive) weight-control regimen including behaviors that most individuals would experience as costly and aversive (e.g. food restriction, purging, excessive exercising) is often attributed to altered reward responding [22, 23] and/or excessive self-regulatory control [15, 17]. A novel theoretical account, the learned industriousness model of AN [16, 24], proposes that excess goal pursuit in the disorder may be attributed to a conditioned reward response to repeated high-effort actions that over time renders effort exertion less aversive and more appetitive. Several lines of indirect evidence suggest that individuals with AN may experience effort as less costly than unaffected individuals including e.g. self-reported feelings of empowerment from restrictive eating [25, 26], a temperamental propensity toward elevated “persistence” and perfectionism [27–29] and high achievement and athleticism [30, 31]. To date, however, no studies have experimentally probed this theory [24, 32].

Effort discounting paradigms offer one means to test of the notion that people with AN experience effort as less costly [24] by describing the computational mechanism underlying motivated behavior as an economic cost-benefit trade-off. While several approaches to both physical and cognitive effort discounting have been developed [13], a common method involves participants making a series of decisions between performing a lower effort task for a smaller reward (usually monetary) or a higher effort task for a larger reward that are incrementally titrated based on the previous choice until the options are equally preferred; thereby enabling estimation of the subjective effort cost (i.e. the decrease in SV of a reward as effort increases). The current study used the COGED paradigm [18, 19] which varies objective cognitive effort using the N-back task to avoid the potential confound of manipulating physical effort in a population where over-exercising is common [33, 34]. Previous fMRI investigations of cognitive effort discounting in non-clinical samples found the SV of chosen effort-reward offers during comparative decision-making to scale with activation in frontoparietal regions associated with cognitive control including posterior medial frontal cortex (pMFC), lateral prefrontal cortex (LPFC) and intraparietal sulcus (IPS) [35–37]. However, because this activation pattern may also reflect control demands associated with decision difficulty (e.g. when options are equally preferred) rather than effort-based cost-benefit valuation per se, it would be advantageous to also investigate SV-related activation associated with single higher-effort/reward offers without invoking deliberation between choices. Following this logic in an fMRI adaptation of COGED used in the current study, Westbrook et al. [19] found in a targeted analysis of activation during contemplation of a single higher-effort/reward offer that SV was encoded in a core valuation network [38] comprised of the ventral striatum (VS) and ventromedial prefrontal cortex (vmPFC). In the current study, we predicted that if AN participants experience effort as less costly than HC, this would be reflected in group differences in SV-related activation in reward-related regions of interest (ROI; VS and vmPFC) in particular during contemplation of single offers. If, however, altered effort-based decision-making in AN is not primarily due to paradoxical reward response to greater effort, but rather exaggerated control [39], we expected that group differences in SV-related activation would be evident in frontoparietal regions in particular during comparative decision-making.

## Methods

### Participants

The final study sample after case-control age-matching and data quality control (Supplementary Information) consisted of 96 women (48 acutely underweight patients with AN and 48 HC) selected from a total of 133 female participants (12–24 years old; 50 AN and 83 HC). Current and past eating disorders were explored in all participants by trained master’s/doctoral level research assistants administering the Structured Interview for Anorexia and Bulimia Nervosa (SIAB-EX; [40]) adapted to DSM-5 criteria. AN participants were admitted to treatment programs at the University Hospital Dresden and assessed within 96 h of beginning nutritional rehabilitation. Inclusion criteria for AN participants included a body mass index (BMI) <10th age percentile or <17.5 kg/m^2^ (if 15.5 years or older) and no sustained recent weight gain (>2.5 kg within the past 4 weeks or 2 kg within the past 2 weeks). HC participants had to have a current BMI of >10th age percentile or 18.5–28 kg/m^2^ (if <15.5 years), be eumenorrhoeic, have no lifetime BMI <10th age percentile or <17.5 kg/m^2^ (if <15.5 years) or history of psychiatric illness as assessed with the Mini International Neuropsychiatric Interview [41]. Several exclusion criteria were applied to both groups including psychotropic medication within the past 4 weeks and medical conditions that influence eating behavior or body weight (Supplementary Information). The Institutional Review Board of the Technische Universität Dresden approved the study and all participants (or their legal guardians) gave written informed consent.

### Clinical measures

Eating disorder-related symptoms were assessed with the Eating Disorders Inventory [EDI-2; [42]. Depressive symptoms were examined using the Beck Depression Inventory–II (BDI-II; [43]. As in previous studies of effort discounting [11, 18, 21], we also administered the Need for Cognition (NFC) questionnaire [44] to assess the trait tendency to engage in and enjoy cognitively effortful tasks. BMI was verified immediately prior to MRI scanning by weighing participants on a calibrated digital scale and measuring height with a stadiometer. BMI standard deviation scores (BMI-SDS; [45, 46]) were computed as an age- and sex-corrected weight-to-height ratio index. Study data were managed using Research Electronic Data Capture (https://www.project-redcap.org/).

### Tasks and procedures

**Fig. 1:**
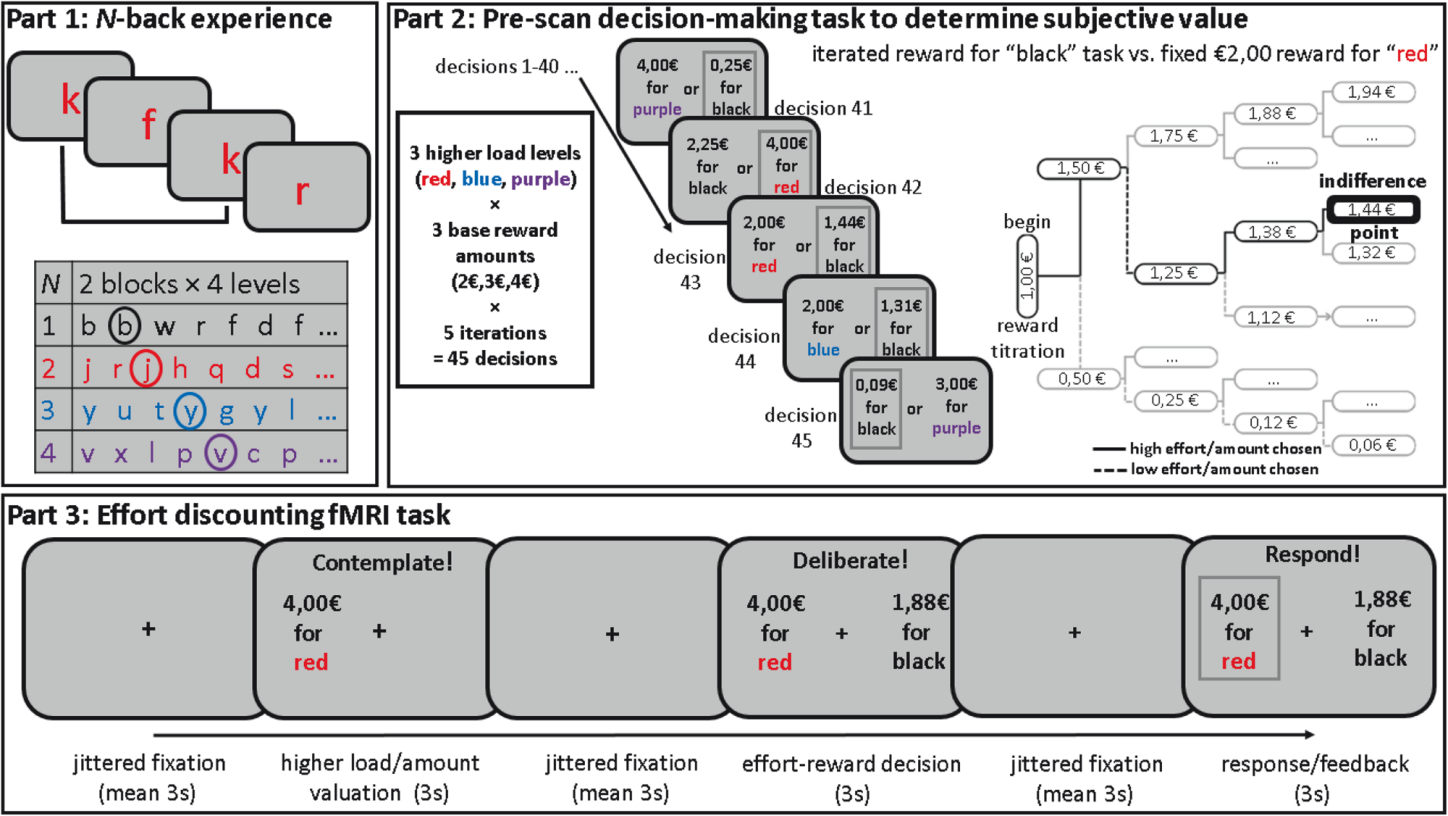
Schematic depiction of the Cognitive Effort Discounting (COGED) paradigm. In Part 1, participants performed two blocks (64 items with 16 targets per block) of each color-coded level of N-back (*N* = 1–4) to familiarize them with the effort demanded by each load level. In Part 2, participants completed an effort-reward decision-making procedure including 45 choices between repeating performance of higher-load N-back for a fixed larger reward or low load 1-back for a variable lower amount. After each choice (confirmed by a gray box surrounding the chosen option), the reward amount offered for the black task was titrated (increased by 50% if the harder option was chosen and vice versa) until participants were indifferent between the options, thereby enabling quantification of effort costliness and the rate at which the subjective value of an offer is discounted by the subjective effort cost (Methods). In Part 3, during fMRI, participants made an additional 90 decisions about effort-reward trade-offs that were individually calibrated based on the indifference points estimated during Part 2 to systematically vary the subjective value of the higher-load offers and decision difficulty (Methods; Supplementary Information).

A modified version of the COGED paradigm was implemented in four parts as in the fMRI study of Westbrook et al. ([19]; Fig. 1). First, in Part 1, “N-back experience”, participants performed two blocks of each level of an N-back task (*N* = 1–4) in order of increasing load, to familiarize them with the effort demanded by each level. N-back task parameters, including the color-coding of load, were as in previous COGED studies (Supplementary Information). After experiencing each N-back level, we administered the NASA Task Load Index (NASA-TLX; [47]) to quantify the associated subjective effort. In Part 2, participants completed an effort discounting calibration procedure, in which they made a series of 45 two-alternative effort-reward decisions between repeating performance of higher-load N-back (*N* = 2, 3 or 4) for a fixed larger base reward amount (€2, €3 or €4) or low load 1-back for a variable lower amount in COGED Part 4 “N-back re-do” (described below). The reward amount for the 1-back option was titrated in a stepwise manner after each choice (over five choices per reward amount- task load pair) until participants were indifferent between the two options (Fig. 1), yielding nine indifference points. The indifference point is important, because it quantifies how costly the participant perceives the more effortful task (i.e. the degree at which the SV of an offer is discounted by the subjective effort cost). To illustrate, if a participant was indifferent between €4.00 for 3-back and €2.13 for 1-back, she perceived the additional effort of the higher-load task to cost €1.87. In this example, we considered the SV of both offers (i.e. €2.13 for the 1-back and €4.00 for the 3-back) to be €2.13. To encourage realistic choices, participants were informed they would make a series of similar decisions during fMRI and receive the reward amount of one of their randomly selected choices, provided they “maintain effort” during performance of “up to two blocks” of the selected N-back in the final COGED Part 4, “N-back re-do”.

In Part 3, participants underwent fMRI while making an additional series of effort-reward decisions that systematically varied according to the individual SV of the higher-load offer and decision difficulty based on the indifference points calculated in the calibration procedure (Part 2). Specifically, over the course of 90 decisions (3 higher-load levels × 3 base reward amounts × 10 repetitions), the SV of the high load offer was manipulated by giving participants choices to perform either one of the higher-load *N*-back tasks (*N* = 2–4) for one of the three base reward amounts (€2, €3, or €4) or the 1-back for a lower variable amount. To promote choices for both options while ensuring that decision difficulty was balanced across participants, the reward amount offered for the 1-back task was varied according to a proximity parameter that specified the distance in SV between the two options, with smaller absolute values being relatively close in SV and positive values indicating higher SV for the 1-back option (Supplementary Information).

Each trial of the fMRI task (Fig. 1) began with the presentation of a single higher-load/reward offer (“valuation” phase) on one side of the monitor (position counterbalanced across trials), followed by presentation of both the higher-load/reward and low-load/reward offers (“decision-making” phase) and ended with a response phase in which participants selected the preferred offer with a left/right index finger button press corresponding to the position of the offer. As in Westbrook et al. [19], the higher-load/reward option was presented alone in the “valuation” phase to isolate activation associated with that offer and the SV thereof, without evoking comparative decision-making in relation to the low load/reward option. To target activation associated with comparative decision-making in the “decision-making” phase [including the SV of the chosen (vs. unchosen) offer] and minimize the influence of response-related processes, participants were instructed to weigh the two options until they reached a decision, but that responses could only be made in the subsequent “response” phase. After the fMRI session, participants consumed a small breakfast, completed COGED Part 4 “N-back re-do” based on one of their decisions, after which they received the actual reward amount as a bonus to base compensation (≈€12/h).

### Behavioral data analysis

On a behavioral level, our primary question was whether the subjective effort costs differed between AN and HC during the pre-scan effort discounting procedure (COGED Part 2). To provide an answer, we fit multilevel models accounting for hierarchical nesting of SVs within participants in R (version 4.3.1 (R Core Team, 2022)) using the lme4 package [48] as in previous COGED studies [18, 20, 21], supplemented by Bayesian modeling with brms [49]. The main model previously demonstrated to distinguish between diagnostic groups [20, 21] included group, N-back load, and the group × load interaction as fixed factors to predict SVs (dependent variable), with intercept and load level effects being allowed to vary by participant. Additional multilevel models explored the predictive effect of reward amount, N-back task performance (signal detection *d*’) and IQ (Supplementary Information). All other analyses of behavioral data were carried out with IBM SPSS (Version 29) software.

### Neuroimaging acquisition, processing and analysis

Structural and functional images were acquired between 8 and 9 am after an overnight fast [50] with a 3T Siemens Trio MRI scanner (Erlangen, Germany) equipped with a 32-channel head coil using standard sequences (Supplementary Information). During fMRI, stimuli were presented using Psychophysics toolbox v3.0.13 (http://www.psychtoolbox.org) and behavioral data were recorded with NordicNeuroLab response grips (NordicNeuroLab, Bergen, Norway). Imaging data were processed in SPM 12 (https://www.fil.ion.ucl.ac.uk/spm/http://www.fil.ion.ucl.ac.uk/spm/) within the Nipype framework [51] including correction for magnetic field inhomogeneities, joint motion and slice timing correction [52], individual co-registration of the functional and structural images, DARTEL normalization [53] to MNI space using the structural images of all participants, spatial smoothing using an isotropic Gaussian kernel with 8 mm FWHM, substraction of physiological noise with CompCor [54] and quality control using artifact detection tools (ART; www.nitrc.org/projects/artifact_detect/; Supplementary Information).

First-level single-subject statistical analysis involved estimation of a GLM including separate binary regressors convolved with a canonical HRF time-locked to the onsets of the two COGED phases of interest: the valuation phase (presentation of only the higher-load/reward offer) and the decision-making phase (presentation of both the higher-load/reward and low-load/reward offers; Fig. 1). Importantly, two single-trial parametric modulators were also included in the design matrix corresponding respectively to the individual SV of the first offer (valuation phase) and the SV of the chosen offer (decision-making phase). Additional regressors of non-interest were participant responses including reaction times, realignment parameters, noise components, and motion/intensity outlier volumes. Our objective was to identify brain regions in which activation during the valuation and decision-making phases correlated with the respective parametric SV modulators, and to test for group differences in this activation pattern. To this end, we ran whole-brain second-level one-sample *t*-tests to identify regions showing activation correlated with the respective SV regressors in all participants, followed by corresponding independent-samples *t*-tests to test for group differences. Additionally, because the easiness/difficulty of comparative decision-making is not orthogonal to the SV of the available options (decisions are usually easier when one option is strongly valued over the other and more difficult when both options are relatively equally preferred), we also explored group differences using a GLM targeting activation associated with the trialwise difference in SV between the chosen and unchosen options (SV_chosen_ - SV_unchosen_) during the decision-making phase. Whole-brain results were familywise error rate corrected for multiple comparisons (*p* < 0.05) with AFNI’s 3dFWHMx and 3dClustSim programs [55] using a cluster-defining voxelwise threshold of *p* < 0.001, unless otherwise noted.

Further exploration of SV-related activation (beta estimates) in a priori ROIs (VS and vmPFC) and regions identified via the whole-brain analyses described above as well as relationships thereof with external variables (EDI-2 total score, BMI-SMS, NFC) is described in the Supplementary Information.

## Results

Demographic and clinical characteristics are summarized in Table 1. As expected, BMI was lower and both eating disorder and depressive symptoms were elevated in AN. IQ estimates were lower in AN compared to HC and therefore accounted for in the main between-group comparisons of behavioral measures of effort discounting and fMRI activation in ROIs. Contrary to the expectation that individuals with AN might self-report a greater tendency to seek out and gain pleasure from cognitively effortful activities, no group difference in NFC was detected.

**Table 1:** Demographic and clinical variables.

	AN	HC	*T*	*P*
Age (years)	15.9 ± 2.2	15.9 ± 2.2	0.06	0.9
IQ	106.2 ± 10.9	114.8 ± 9.3	4.1	<0.001
Min. lifetime BMI (kg/m^2^)	14.6 ± 1.5	20.6 ± 1.7	17.7	<0.001
Current BMI (kg/m^2^)	21.1 ± 1.9	21.6 ± 1.8	18.3	<0.001
Current BMI-SDS	−3.2 ± 1.2	0.02 ± 0.6	17.0	<0.001
Age of onset (years)	14.8 ± 1.8	NA	NA	NA
Duration of current episode (months)	13.7 ± 12.7	NA	NA	NA
BDI-II	25.3 ± 10.2	4.8 ± 5.4	12.2	<0.001
EDI-2 total	217.9 ± 47.9	133.9 ± 28.7	10.2	<0.001
Drive for thinness	29.63 ± 10.2	11.1 ± 4.1	11.7	<0.001
Body dissatisfaction	39.2 ± 10.2	20.4 ± 8.2	9.9	<0.001
Bulimia	12.3 ± 5.8	9.4 ± 2.2	3.3	<0.002
NFC	14.9 ± 22.8	14.3 ± 21.3	0.13	0.9

Thirty-seven patients with anorexia nervosa (AN) were of the restrictive subtype (77%) and 11 were of the binge/purge subtype (23%). Nine patients with AN were diagnosed with of one or more active comorbid psychiatric disorders (3 with depression, 3 with anxiety disorders, 3 with obsessive-compulsive disorder). Data are presented as mean ± standard deviation.

*BDI-II* Beck Depression Inventory–II, *BMI* body mass index, *BMI-SDS* body mass index standard deviation score, *EDI-2* Eating Disorder Inventory–2, *HC* healthy control, *NA* not applicable.

### N-back experience: effect of cognitive load on performance and subjective effort costs

Analysis of N-back experience (COGED Part 1) performance (signal detection *d*’ values) confirmed the detrimental effect of load, but groups did not differ in this respect (group × load; Supplementary Information). However, *d*’ values were generally higher in AN (*p* < 0.05), indicative of overall superior performance (Supplementary Fig. S1). Despite generally better N-back performance in AN, NASA-TLX ratings showed that patients experienced the task as generally more “frustrating”, perceived 4-back as particularly demanding (both *p* < 0.05), and tended to underestimate their performance (*p* < 0.1; Supplementary Fig. S2).

### Behavioral pre-scan effort discounting procedure

**Fig. 2:**
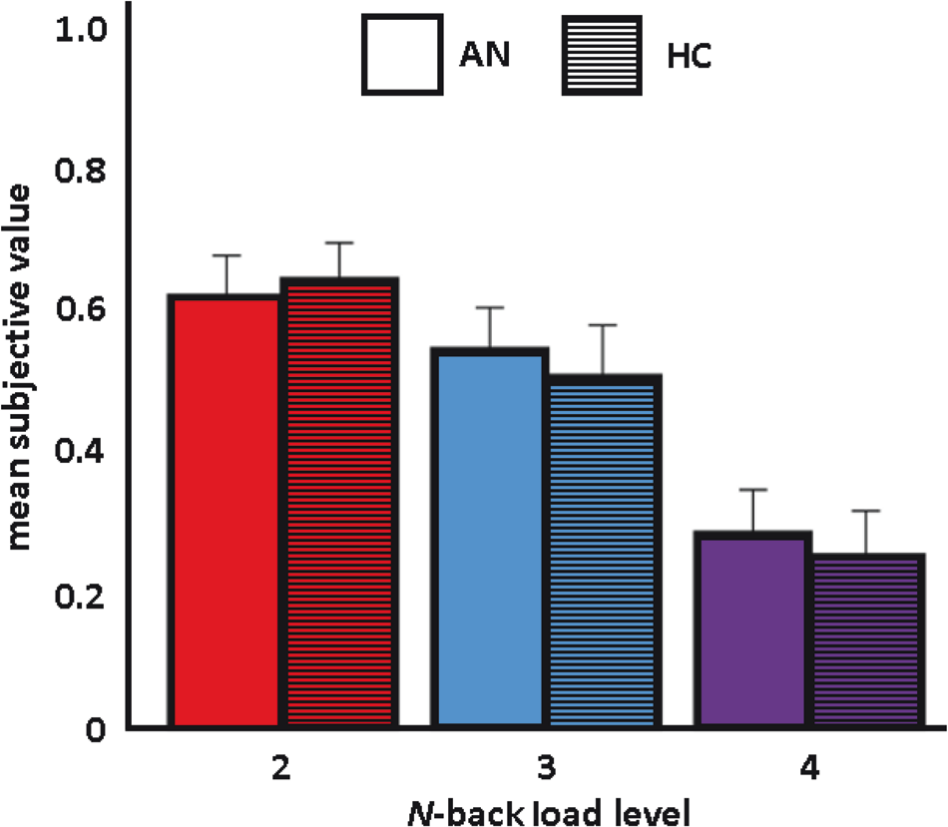
Main behavioral results. Decreasing mean subjective value of rewards offered for higher-load N-back estimated from indifference points (averaged across base reward amounts; ±SEM) calculated from choices made during the pre-scan effort-reward decision-making procedure (COGED Part 2) is plotted by diagnostic group; illustrating the increasing subjective cost as a function of increasing cognitive load, but overall comparable cognitive effort discounting between groups. AN anorexia nervosa, HC healthy control.

Choice behavior during the pre-scan effort-reward decision-making procedure (COGED Part 2) showed that participants discounted rewards offered for higher N-back levels as expected, with SV decreasing for each load increase in both groups (Fig. 2). Multilevel model analysis confirmed the predictive effect of N-back load on the SV of chosen offers (*β* = −0.16, *t* = 15.5, *p* < 0.001), but neither group (*β* = −0.009, *t* = 0.48, *p* = 0.6) nor the group × load interaction (*β* = −0.008, *t* = 0.80, *p* = 0.4) was a significant predictor of SV in the main model. Additional models revealed that N-back performance (*d*’) was also predictive of SV, but the group predictor had no significant effect and did not interact with any other variable (Supplementary Table S1). Supplementary Bayesian modeling underlined the lack of a group difference in effort discounting (all BF_01_ ≥ 68.49; Supplementary Table S2). Group comparisons of two related summary measures of effort costliness, specifically, area under the curve (AUC) [56] and the proportion of choices for the higher-load/reward option, also revealed no differences (both *t*_94_ < 0.6; both *p* > 0.3; Supplementary Information). Moreover, no significant correlations were found in exploratory analysis of relationships with NFC or AN-related clinical variables in patients (EDI-2 total, BMI-SDS; all *p* > 0.18).

### Effort discounting fMRI session

As in Westbrook et al. [19], behavior during the fMRI session was not a primary dependent variable given the manipulation of effort-reward offers to balance choice preferences and control decision difficulty (Methods, Supplementary Information). Validating the manipulation, however, the pre-scan effort discounting procedure accurately identified indifference points and biased choice preferences during the fMRI session accordingly (Supplementary Fig. S3).

### Whole-brain analyses

Activation during the COGED Part 3 valuation phase (Fig. 1) correlated positively with trialwise SV of the first higher-load/reward offer in bilateral regions of visual cortex in the complete sample (Supplementary Fig. S4, Supplementary Table S3), but no group differences reached significance. However, exploration of first offer SV-related activation within each group separately confirmed greater engagement of striatal reward circuitry in HC as expected ([19]; Supplementary Fig. S5, Supplementary Table S4).

**Fig. 3:**
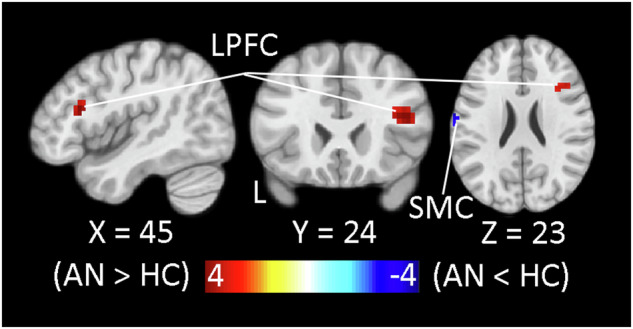
Group differences in activation associated with subjective value representation during decision-making. Greater chosen offer SV-related activation during the decision-making phase of COGED part 3 in AN > HC (warm colors) in a region of the right inferior frontal gyrus in lateral prefrontal cortex (LPFC; 42, 24, 19; *Tmax* = 4.20; *k* = 33) and in AN < HC (cool colors) in a region of left primary sensorimotor cortex (SMC; −66, −26, 27; *Tmax* = 4.12; *k* = 71) as revealed by trialwise parametric analysis is shown at on selected slices of the MNI152 template at a voxelwise threshold of *p* < 0.001 (whole-brain corrected, *p* < 0.05).

During the decision-making phase (Fig. 1), activation correlated positively with the SV of the chosen offer in the entire sample in regions involved in reward processing (striatum including VS), cognitive control and decision-making (pMFC, bilateral LPFC, anterior insula, and posterior parietal cortex including IPS) as expected [36], while a negative correlation was evident in vmPFC (Supplementary Fig. S6, Supplementary Table S5). More important with respect to the hypothesis of altered effort-based decision-making in AN, chosen offer SV-related activation significantly differed between groups: while HC showed greater activation as a function of SV in a region of left primary sensorimotor cortex likely involved in response preparation, AN showed greater SV-related activation in a region of right LPFC broadly implicated in integrating motivation and cognitive control [57–59]; (Fig. 3).

**Fig. 4:**
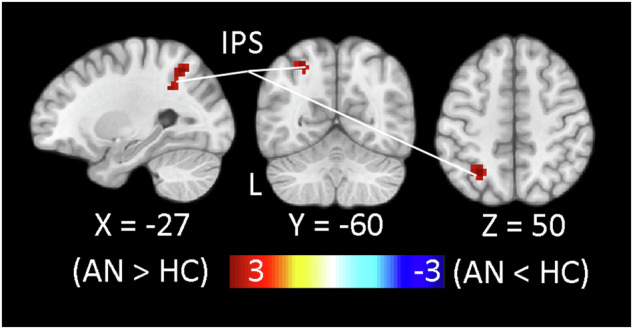
Group differences in activation related to the decision easiness/difficulty. Greater activation in AN > HC during the decision-making phase of COGED part 3 as a function of the difference in SV between the chosen and unchosen options (SV_chosen_ - SV_unchosen_) in a region of the left superior intraparietal sulcus (IPS; −27, −60, 50; *T*max = 3.90; *k* = 51) as revealed by trialwise parametric analysis is shown on selected slices of the MNI152 template at a voxelwise threshold of *p* < 0.001 (whole-brain corrected, *p* < 0.05). The group difference in this relationship was bilateral at a more lenient voxelwise threshold (Supplementary Fig. S8).

Because the difficulty of cost-benefit calculations when choosing between the two effort-reward options is dependent on their SV (decisions between alternatives tend to be easier if we have a strong preference for one and more difficult if they are equally valued), we also explored activation during the decision-making phase associated with the trialwise difference in SV between the chosen and unchosen options (SV_chosen_ - SV_unchosen_). In the entire sample, results revealed a positive correlation indicating greater activation on trials in which decisions were easier (i.e. the difference in SV between the alternatives was large) in some of the same regions identified by the basic analysis of chosen offer SV-related decision-making activation in the in the whole sample (bilateral anterior insula, left LPFC) and posterior midcingulate cortex (Supplementary Fig. S7; Supplementary Table S6; see also Supplementary Fig. S6). In contrast, a negative correlation indicating greater activation on trials in which decisions tended to be more difficult (i.e. the difference in SV between the alternatives was relatively small) was revealed in a region of right IPS (Supplementary Fig. S7, Supplementary Table S6). More importantly, group comparison revealed greater activation in AN compared to HC when the difference in SV between effort-reward alternatives was larger in a corresponding region of left IPS (Fig. 4). This group difference in IPS was bilateral at a more lenient voxelwise threshold (*p* < 0.01; whole-brain corrected *p* < 0.05; Supplementary Fig. S8), but no other group differences emerged. Together, these findings suggest that AN group recruitment of frontoparietal control regions during effort-based decision-making may not merely reflect “working harder” to tackle difficult decisions. Particularly the group difference in the IPS suggests that individuals with AN tend to execute control even when they preferred the chosen option to a greater degree, i.e. on trials when intensive deliberation would not be needed.

### ROI analyses

Closer inspection of SV-related activation (beta estimates) extracted from the a priori-defined VS and vmPFC ROIs during both the valuation and decision-making phases confirmed the absence of any group differences in valuation circuitry (all *t* < 0.15; all *q* > 0.14; which were further supported by supplementary Bayesian analyses (all BF_01_ ≥ 2.5; Supplementary Information). Results of further analysis of SV-related activation during decision-making in the identified LPFC and intraparietal regions (Figs. 3 and 4) mirrored the observed group differences as expected (both *t* > 3.6; both *p* < 0.001) and showed that they could not be explained by the group difference in IQ (Supplementary Information). However, no relationships between SV-related frontoparietal activation and external variables (EDI-2 total, BMI-SMS, NFC) survived multiple comparisons correction (all *r* < 0.31; all *q* > 0.11).

## Discussion

This study employed a cognitive effort discounting paradigm (COGED; [18, 19]) in combination with fMRI to adjudicate between current theories of AN that differentially emphasize altered reward responding [22, 23] and exaggerated control [15, 17] as key factors underlying disorder-characteristic extreme weight-control behavior and excess goal pursuit [16]. Contrary to our motivating hypothesis, we found no behavioral evidence indicating that effort is less costly in AN as assessed either objectively with COGED or via self-report with the NFC scale. In fact, some self-report evidence from NASA-TLX ratings of the N-back experience phase (elevated frustration scale scores, greater perceived task demand particularly for the 4-back task) seemed to suggest that AN participants might experience effort as *more* costly and aversive than HC. However, generally in line with our hypotheses and the learned industriousness theory of AN [24], these findings might also reflect heightened goal-directedness in AN and associated feelings of discontent when high personal standards are not met. On a neural level, some evidence hinted that individuals with AN might not recruit striatal reward regions as a function of effort-reward valuation to the same extent as HC, but no group differences in either VS or vmPFC reached significance. Instead, group differences observed in both behavior and fMRI activation were collectively more suggestive of excessive control in AN. First, N-back experience performance was generally superior in AN relative to HC, indicative of greater effort exertion or motivation. Although this finding (and the associated aforementioned group differences in NASA-TLX ratings) is tangential to the question whether effort-based decision-making is altered in AN, it is compatible with diverse previous findings of overcontrolled task performance in the disorder [60–65]. More directly related to the study hypotheses, while both groups recruited broad frontoparietal control regions as a function of chosen offer SV during comparative decision-making, AN participants recruited LPFC to a greater extent. Given the role of the LPFC in integrating motivation and cognitive control and value-based decision-making [57, 66], this finding suggests that the AN group engaged in more controlled deliberation, particularly for highly valued choices. Complementing this finding, while activation of the IPS during comparative decision-making scaled with decision difficulty in both groups (i.e. it was stronger when the difference in SV between the two effort-reward offers was smaller), the AN group recruited this region to a greater degree than HC, even when decisions were comparatively easy and the SV of the chosen option was sizably greater than that of the unchosen one. Together, these findings offer at least two important new insights on motivated behavior in AN. First, the lack of group differences in either behavioral measures of effort discounting or neural indices of effort-reward valuation (especially SV-related activity in VS and/or vmPFC) suggest that altered reward responding in AN [22, 23] may not generalize to situations involving effort-based decision-making [16]. Second, and relatedly, while our findings lend no clear support for the notion that individuals with AN might experience effort as less costly [24], the observations indicative of exaggerated control and effort investment (superior N-back performance and associated NASA-TLX ratings; increased SV-related frontoparietal activity) suggest that they might actually “go the extra mile”, even in the absence of particular cognitive demands (such as when performing low-load N-back or deciding between strongly preferred and nonpreferred effort-reward options) or disorder-related rewards.

Behavioral studies of effort discounting have consistently observed a diminished willingness to exert effort for rewards in psychiatric disorders typically characterized by amotivation [20, 21, 67–70]. In line with these findings, fMRI studies in schizophrenia have found clinical amotivation to correlate inversely with SV-related VS activation [71, 72]. However, findings have been less clear in other conditions also commonly characterized by motivational impairments. For example, studies in ADHD have not found steeper discounting [73, 74] and some evidence in other conditions has even suggested an unexpected greater willingness to engage in effortful tasks [75, 76]. Although no known studies in clinical eating disorders exist [16, 24, 32], eating pathology in transdiagnostic samples has been linked to less cognitive effort avoidance [77] and preliminary evidence suggests that binge-eating symptoms might be related to the willingness to work (physically) for food rewards [78]. To better understand why we did not observe shallower effort discounting in AN and/or elevated SV-related activation in reward-related brain circuitry, future research might employ tasks involving actual effort expenditure [7] with e.g. different types of disorder-relevant effort demands [79] and/or rewards [22]. It may also be advantageous to better control for task difficulty [80] e.g. by evaluating effort choice independently from performance and exploring of the process of learning the value of effort [81]. Experimental approaches accounting for e.g. individual differences in preferences for effortful tasks [82] and that positively affect task engagement by design [83] might also add perspective on the hypothesized greater willingness to exert effort in AN. We speculate that individuals with AN, who are often perfectionistic and have heightened feelings of ineffectiveness [84], as exemplified here in NASA-TLX ratings indicating elevated frustration with N-back and the tendency to underestimate performance, might be more willing to engage in effortful tasks in which they feel more competent.

The fMRI findings of increased activation in LPFC and IPS in AN during effort-based decision-making add to studies that have observed hyperactivation of frontoparietal control regions in AN samples in other functional domains including e.g. at rest [85], during set-shifting [86], reward anticipation [87], emotion regulation [88, 89] and processing of disorder-related stimuli [90, 91]. Most notably in relation to the current study of effort discounting, fMRI studies of delay discounting have found increased LPFC activation in weight-restored individuals with a history of AN [92, 93], suggestive of a trait-like tendency of heightened self-control. However, similar to the current behavioral findings of intact effort discounting, several delay discounting studies did not find an increased capacity to delay reward in AN [94, 95]. Regarding the specific frontoparietal regions in which SV-related chosen (vs. unchosen) offer activation was elevated in AN, a recent brain stimulation study using an analog COGED task found the same LPFC region identified in AN (albeit in the left hemisphere) to be casually involved in the motivation to exert effort [96]. Similar to the current findings, the identified IPS region, part of the dorsal attention network involved in voluntary visual orienting [97], has been linked to cost–benefit calculation (between peripherally presented choice alternatives) during value-based decision-making [98]. The observed hyperactivation in these regions in AN on trials involving highly-valued chosen (vs. unchosen) options (which tend to be easier decisions) is generally consistent with the clinical presentation of overcontrol in AN [99] and the related behavioral and neuroscientific findings noted above. These findings provide further justification for continued evaluation of treatment approaches targeting associated difficulties in the disorder (cognitive/behavioral inflexibility, intolerance of uncertainty, emotional inhibition [100]).

Our findings should be considered in light of some noteworthy study limitations in addition to those mentioned above. First, although the COGED paradigm produced an expected pattern of effort discounting, it did not differentiate between groups on a behavioral level as predicted. This should not be overinterpreted, however, because the type of cognitive effort demanded by the N-back task may not test the type of control that individuals with AN seem to experience as less costly (or more rewarding) in their everyday lives (e.g. inhibiting approach motivation and restrictive eating). Furthermore, as we have previously noted in relation to the overall inconsistent findings on delay discounting in AN [95], we speculate that while discounting tasks may readily capture “too little” control (e.g. amotivation or impulsivity), it may be more difficult for them to identify overcontrolled choice behavior. Second, and relatedly, the use of an external (monetary) value metric detracts from assessing the intrinsic value of effort and future research may benefit from modeling effort willingness in the absence of reward or controlling for reward effects [6, 81, 101, 102]. Third, although the fMRI adaptation of COGED distinguished between groups in a manner consistent with the notion of exaggerated control in AN, the overall pattern of neural responding (including e.g. the lack of group differences in reward regions during effort valuation) may have been influenced by habituation effects given that participants already made a series of effort-reward decisions in COGED Part 2 before scanning). Fourth, despite pair-wise case-control age-matching, the AN group had a lower IQ compared to HC. Although sensitivity analyses indicated that IQ had no impact on the main group differences in either behavior or fMRI activation, we cannot entirely rule out any possible influence or that group differences may simply reflect some other state-based compensatory mechanism due to e.g. chronic starvation effects. Last but not least, our findings may only apply to young women in the acutely underweight state and may not generalize to older individuals (including men) with a longer duration of illness or persist after weight restoration, as would be expected if exaggerated control were a disorder-defining trait.

In summary, this first study of effort discounting in AN contributes to the understanding of effort-based decision-making and motivated behavior in the disorder by highlighting the role of excessive control. While our findings suggesting that individuals with AN may not experience effort as less costly (or more rewarding) than HC need to be interpreted cautiously due to the limitations noted above, the data indicate that people with the disorder seem to invest it more than needed both when performing demanding cognitive tasks and when making decisions about effort-reward trade-offs. This pattern of results stands in stark contrast to that of diminished effort willingness commonly observed in many mental health conditions characterized by amotivation and provides initial indirect support of the learned industriousness model of AN [22]. Although the study results need to be confirmed and extended in independent AN samples, they underline the need to develop therapeutic approaches that help channel the astonishing (albeit maladaptive) achievement orientation that paradoxically constrains the lives of those with the disorder into the pursuit of more beneficial mental and physical goals.

## Supplementary information

Supplementary Information

## Data Availability

The data and analysis code that support the findings of this study are available upon reasonable request.
