# Supplementary material for: Exaggerated frontoparietal control over cognitive effort-based decision-making in young women with anorexia nervosa
Source: Mol Psychiatry. 2024 Aug 28;30(3):861–9. doi: 10.1038/s41380-024-02712-4 (PMC11835750; doi:10.1038/s41380-024-02712-4)
Supplement: Supplementary file 1 — Supplementary Information [file 41380_2024_2712_MOESM1_ESM.docx]

# Exaggerated frontoparietal control over cognitive effort-based decision-making in young women with anorexia nervosa

# Supplementary Information

**Supplementary Methods**

**Participants**
To minimize potentially confounding (neuro-)developmental effects and optimize between-group comparisons, HC were pseudorandomly selected from an oversampled pool of HC participants to pairwise age-match AN participants using a mathematical algorithm [1] with a maximum difference of 0.5 years in each pair. HC were oversampled to enable case-control age-matching in other studies focused on a predominately adult cohort of long-term weight-recovered individuals with a history of AN. Prior to age-matching, the fMRI data of 4 participants were excluded due to technical issues: a hardware error that interfered with response recording (2 AN) and susceptibility artifacts (2 HC).

Additional exclusion criteria applied to both groups included consumption of any psychotropic substance within the past 4 weeks (expect for SSRIs in AN; *n* = 0 in the current sample), any history of organic brain syndrome, schizophrenia, bipolar disorder, or psychosis not otherwise specified; current inflammatory, neurologic, or metabolic disorders. Further, participants were not included if they were pregnant or breast feeding, had anemia, or if their estimated IQ was < 85.

We collected demographic information regarding household occupational status with which we could estimate a measure of socioeconomic status (SES) [2] in the majority of AN (*n* = 45) and HC (*n* = 41) participants.

**N-back task**

Following brief verbal and written instruction, each experimental session began with “*N*-back experience” (COGED Part 1). N-back experience involved performance of 2 blocks of each of the 4 color-coded load levels (black = 1-back, red = 2-back, blue = 3-back, purple = 4-back), each comprised of 64 items (consonants presented centrally in 120-point Courier New font), of which 16 were targets. Stimuli were shown for 1.5s separated by an interstimulus interval of 1s, during which a fixation cross (24-point font) was presented. Lures (defined as identical items within N+2, but not exactly *N*, positions following the last presentation) were included in stimulus lists to increase the difficulty of each load level: eight for N = 1, six for N  =  2, five for N  = 3, and three for N  =  4. Feedback informing participants about performance quality (% of targets and % non-targets correct) was presented at the end of each block. To encourage task engagement and prevent participants from overemphasizing target vs. non-target responding, feedback of “Good job! Carry on!” was also presented if both scores were above 50% or “Please try harder!” otherwise.

**Effort discounting fMRI task (COGED Part 3): calibration of effort-reward offers to balance choice preference and control decision difficulty**

As in the COGED fMRI study of Westbrook et al. [3], choice preference and decision difficulty were manipulated by varying the reward amount offered for the 1-back task according to a proximity parameter (*γ*) that specified the estimated SV between the two options, with smaller absolute values being relatively close and positive values indicating higher SV for the low load 1-back option. In detail, *γ* quantifies the proportion difference between indifference (*γ* = 0) and the bounds: e.g., *γ* = −0.1 indicates that the offer for the 1-back task is 10% below the indifference point, whereas *γ* = 0.6 indicates that the offer for the 1-back task is above the indifference point by 60% of the difference between the indifference point and the reward amount offered for the higher load N-back task. There were 18 trials for each proximity value from the set {−0.4, −0.1, 0.2, 0.6} yielding 72 regular trials, and 9 trials each from the set {−1.0, 1.0} yielding 18 “catch” trials; respectively equally distributed across the 3 higher load offers. The “catch” trials involve obvious choices: either the 1-back task was associated with a 0€ reward (*γ* = −1.0) which should strongly bias choices in favor of the higher load/reward offer, or the same amount was offered for both tasks (*γ* = 1.0) which should strongly bias choices in favor of low load 1-back task.

**Neuroimaging acquisition parameters**

T1-weighted structural brain scans were acquired with a 3D magnetization-prepared rapid acquisition gradient echo (MP-RAGE) sequence (176 slices, TR = 1.9 s, TE = 2.26 ms, FOV = 256 × 224 × 176 mm^3^, 1 mm^3^ isotropic voxel size, flip angle = 9°). Functional images were acquired during COGED part 3 using a gradient-echo T2^∗^-weighted EPI sequence was used (TR = 2070 ms, TE = 25 ms, flip angle 80°). In total, 705 volumes were obtained (36 axial slices oriented 15° clockwise to the AC-PC line, slice thickness = 3.2 mm, interslice gap = 20%, FoV = 192 × 192 mm, in-plane resolution 3 × 3 mm, voxel size 3 × 3 × 3.2 mm^3^, flip angle = 80°).

**fMRI quality control**

Pre-processing of the functional data was otherwise identical to the pipeline employed in a recent fMRI study from our lab [4]. Prior to statistical analysis, we evaluated data quality by manual inspection and using artifact detection tools to identify volumes with excessive movement (> 2 mm in any direction) and/or intensity outliers (> 3 SD from the mean of the time series). The AN and HC groups did not differ either in the mean (SD) number of volumes per participant with motion outliers [AN: .69 (1.7); HC: 1.9 (5.0); *t*_94_=. 1.7; *p* = .1], intensity outliers [AN: 9.9 (4.1); HC: 9.9 (5.1); *t*_94_ = .04; *p* = .5] or combined motion and intensity outliers [AN: .3 (.6); HC: .4 (1.0); *t*_94_=.37; *p* = .7]. The indices of the outlier volumes were used to create nuisance covariates in the first level GLMs described in the main article.

**Supplementary Results**

**N-Back experience (COGED Part 1) performance**

Technical errors resulted in data being improperly saved for one or more blocks of *N*-Back Experience in 3 AN and 4 HC participants. In the complete data from the remaining 45 AN and 44 HC participants, performance (signal detection *d*’ values) generally declined with increasing task load in both groups (Fig. S1) as expected. A 2 (group) × 4 (*N*-back load) repeated measures ANOVA of *d*’ values confirmed the expected detrimental effect of load (*F*_3,261_ = 317.17; *p* < .001), but performance did not differ as a function of load between the groups (*F*_3,261_=1.98; *p* = .12). However, indicative of overall more accurate performance in patients relative to controls, mean *d*’ values in AN (2.4 ± 0.8 SEM) were generally higher than in HC (2.0 ± 0.8 SEM; *F*_1,87_=5.4; *p* = .022; Fig. S1). A follow-up ANCOVA covarying for IQ confirmed a significant effect of IQ (*F* = 7.7; *p* = .007), but the general group difference in performance also remained significant (*F* = 10.7; *p* = .002).


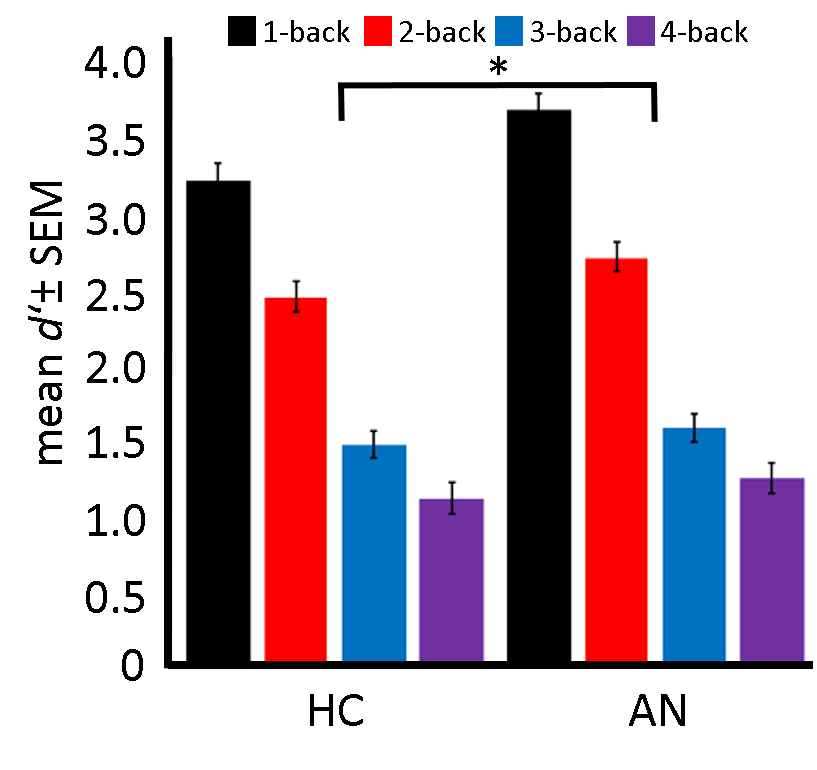


Fig. S1. Objective N-back task load effects on performance. *N*-back experience performance (mean signal detection *d*' values) is plotted by group and *N*-back load level. * signifies the significant main effect of group (*p* < .05) indicating generally more accurate performance in AN relative to HC.

**NASA-TLX ratings of N-back experience (COGED Part 1)**

As in previous COGED studies [5, 6], we administered the NASA Task Load Index (NASA-TLX) [7] after participants completed each N-back load level to quantify the associated subjective effort costs. Fig. S2 summarizes the NASA-TLX Likert scale ratings (range: 1-10) averaged across the six subscales (mental demand, physical demand, temporal demand, performance, effort, frustration). In line with the objective N-back experience performance data (signal detection *d*’ values; Fig. S1), subjective ratings generally increased with increasing N-back load in both groups. A 2 (group) × 4 (N-back load) × 6 (subscale) repeated measures ANOVA of NASA-TLX ratings confirmed significant main effects of both load and subscale (both *F* > 66.9; both *p* < .001) and showed that the groups differed as a function of each (group × load: *F*_3,282_ = 3.8; *p* = .011; group × subscale: *F*_5,470_ = 3.7; *p* = .003). Post-hoc *t*-tests determined that while the group × load interaction could be largely explained by higher ratings (i.e. greater perceived task demand) in the AN group specifically for the 4-back task (*t*_94_ = 2.64; *p* = .02), the group × subscale interaction could be explained by AN group experiencing significantly greater “frustration” (*t*_94_ = 2.64; *p* = .018) with the N-back task in general, together with numerical trends for higher perceived overall “effort” (*p* = .09) and underestimation of own “performance” (*t*_94_ = 2.64; *p* = .075).


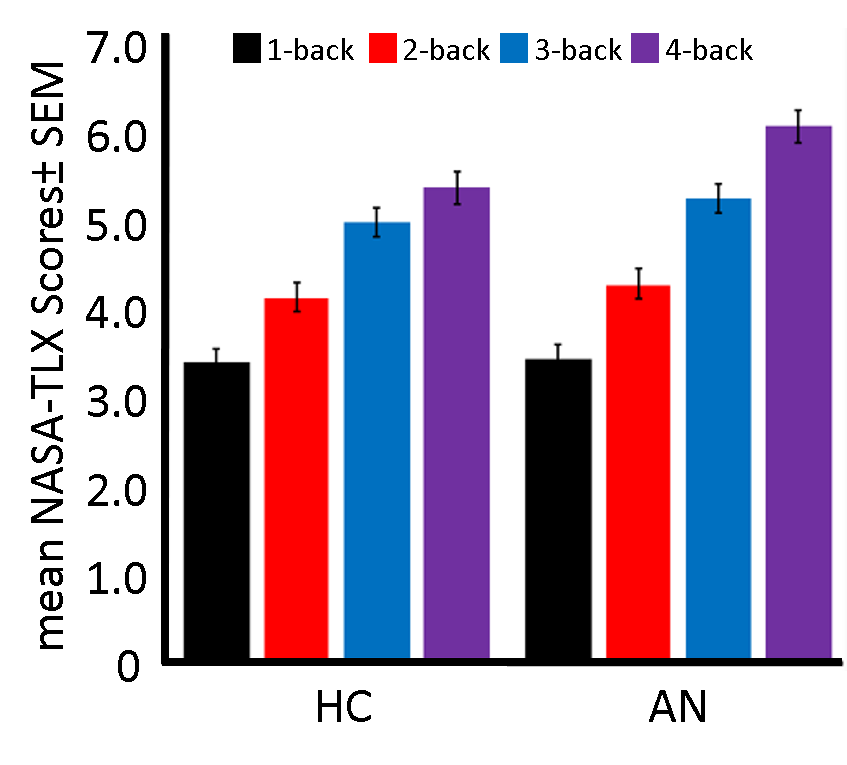


Fig. S2. Subjective N-back task load effects. Mean NASA-TLX scores averaged across all subscales are plotted by group and N-back load level. A detailed description of group differences as a function of both N-back load level and NASA-TLX subscale is provided in the preceding paragraph.

**Pre-scan behavioral effort discounting calibration procedure (COGED Part 2) multilevel model analysis**

Following previous COGED studies [5, 6, 8], we fit several different multilevel models in R software [9] version 4.3.1 using the lme4 package [10] to explore the predictive effect of relevant variables [N-back load, reward amount, N-back performance (*d*’ values), IQ], in addition to diagnostic group, on the SV of chosen offers during the pre-scan behavioral effort discounting procedure (COGED Part 2; Fig. 1). The results are summarized below in Table S1. Likelihood-ratio test was used to compare models relative to the most parsimonious one as determined by previous COGED studies (Model I). Note that neither diagnostic group nor any group × condition interaction was significant in any model. Note also that although the model adjusting for N-back performance (Model IV) could be considered superior to Model I despite added complexity, we report Model I in the main article for comparability with previous studies that reported differences between diagnostic groups using this model [5, 8].

**Table S1. Pre-scan behavioral effort discounting procedure (COGED Part 2) multilevel model results**

| Parameter | Estimate | Standard  error | *T* | *p* | | Model comparison |
| --- | --- | --- | --- | --- | --- | --- |
| **Model I “load” -** | | | | | | |
| Intercept | 0.48 | 0.02 | 26.82 | < .00001 |  | |
| Load | -0.16 | 0.01 | -15.55 | < .00001 |  | |
| Group | -0.01 | .02 | -0.48 | 0.63 |  | |
| Group × load | -0.01 | .02 | -0.79 | 0.43 |  | |
| **Model II “load + IQ” χ2 = 0.30 p = 0.86** | | | | | | |
| Intercept | 0.48 | 0.02 | 24.56 | < .00001 |  | |
| Load | -0.16 | 0.01 | -15.55 | < .00001 |  | |
| IQ | -0.002 | 0.02 | -0.10 | 0.92 |  | |
| Group | -0.01 | 0.02 | -0.39 | 0.70 |  | |
| Group × IQ | -0.01 | 0.02 | -0.55 | 0.58 |  | |
| Group × load | -0.01 | 0.01 | -0.80 | 0.43 |  | |
| **Model III “load + amount” χ2 = 1.74 p = 0.42** | | | | | | |
| Intercept | 0.48 | 0.02 | 26.82 | < .00001 |  | |
| Load | -0.16 | 0.01 | -15.5 | < .00001 |  | |
| Amount | -0.01 | 0.01 | -0.86 | 0.38 |  | |
| Group | -0.01 | 0.02 | -0.48 | 0.63 |  | |
| Group × amount | -0.01 | 0.01 | -0.99 | 0.32 |  | |
| Group × load | -0.01 | 0.01 | -0.80 | 0.43 |  | |
| **Model IV “load + performance” χ2 = 6.06 p = 0.048** | | | | | | |
| Intercept | 0.48 | 0.02 | 27.37 | < .00001 |  | |
| Load | -0.14 | 0.01 | -10.43 | < .00001 |  | |
| Performance | 0.04 | 0.02 | 2.48 | 0.01 |  | |
| Group | -0.01 | 0.02 | 0.35 | 0.73 |  | |
| Group × performance | -0.01 | 0.02 | -0.87 | 0.39 |  | |
| Group × load | -0.01 | 0.01 | -0.96 | 0.34 |  | |

Our motivating hypothesis was that individuals with AN would differ from HC in cognitive effort discounting and that this would be expressed in a group × load interaction in the multilevel models described above. Specifically, we hypothesized that the discounting rate across increasing levels of effort would be less steep and relatively flat (i.e. lower) in the AN group compared to the HC group. Contrary to this hypothesis, however, the results obtained with traditional null hypothesis significance testing using lme4 as described above (summarized in Fig. 1 and Table S1) indicated that the groups did not differ in this respect. To supplement these results and provide more evidence in support of the lack of a group difference in effort discounting, we used the brms package [11] to calculate Bayesian multilevel models equivalent to those calculated with frequentist statistics using lme4 (see above, Table S1) while adopting the same procedures as previously applied to COGED data in a recent study from Aschenbrenner et al. [12]. Specifically, for each of the 4 models, SV was modelled using a Student’s T distribution with flat priors for both the intercept and beta coefficients and 4 chains of 4000 iterations. In all cases, the results (mean posterior probability estimates and 95% credible intervals (CI); summarized in Table S2) mirrored those obtained with lme4 and model convergence was excellent with Rhats = 1.00 and effective sample sizes of ~ 3000 or more for all parameters. Most importantly, CIs for the group × load interaction all included zero, underlining non-significance. Additionally, to quantify evidence supporting the null hypothesis, we calculated Bayes factors (BF_01_) representing the likelihood of the null model (no group × load parameter) versus the alternative model including a group × load interaction. In all cases, BF_01_ was ≥ 68.49 indicating very strong evidence in favoring the null relative to the alternative hypothesis.

**Table S2. Pre-scan behavioral effort discounting procedure Bayesian multilevel model results**

|  |  | | |  | | | 95% Credible Interval | | |  |
| --- | --- | --- | --- | --- | --- | --- | --- | --- | --- | --- |
| Parameter | | Estimate | | | | Standard  error | | Lower | Upper | BF_01_ |
| **Model I “load”** | | | | | | | | | |  |
| Intercept | | | 0.48 | | 0.02 | | 0.44 | | 0.52 |  |
| Load | | | -0.16 | | 0.01 | | -0.19 | | -0.14 |  |
| Group | | | -0.01 | | 0.02 | | -0.04 | | 0.03 |  |
| Group × load | | | -0.01 | | 0.01 | | -0.03 | | 0.01 | 68.49 |
| **Model II “load + IQ”** | | | | | | | | | |  |
| Intercept | | | 0.49 | | 0.02 | | 0.44 | | 0.53 |  |
| Load | | | -0.16 | | 0.01 | | -0.19 | | -0.14 |  |
| IQ | | | -0.00 | | 0.02 | | -0.05 | | 0.04 |  |
| Group | | | -0.02 | | 0.02 | | -0.06 | | 0.03 |  |
| Group × IQ | | | -0.01 | | 0.02 | | -0.05 | | 0.04 |  |
| Group × load | | | -0.01 | | 0.01 | | -0.03 | | 0.02 | 3125 |
| **Model III “load + amount”** | | | | | | | | | |  |
| Intercept | | | 0.48 | | 0.02 | | 0.44 | | 0.52 |  |
| Load | | | -0.17 | | 0.01 | | -0.19 | | -0.14 |  |
| Amount | | | -0.01 | | 0.01 | | -0.02 | | 0.01 |  |
| Group | | | -0.02 | | 0.02 | | -0.06 | | 0.02 |  |
| Group × amount | | | -0.01 | | 0.01 | | -0.03 | | 0.01 |  |
| Group × load | | | -0.01 | | 0.01 | | -0.03 | | 0.02 | 4166.67 |
| **Model IV “load + performance”** | | | | | | | | | |  |
| Intercept | | | 0.48 | | 0.02 | | 0.44 | | 0.52 |  |
| Load | | | -0.14 | | 0.01 | | -0.17 | | -0.11 |  |
| Performance | | | 0.04 | | 0.02 | | 0.01 | | 0.07 |  |
| Group | | | -0.01 | | 0.02 | | -0.05 | | 0.02 |  |
| Group × performance | | | -0.01 | | 0.02 | | -0.04 | | 0.02 |  |
| Group × load | | | -0.01 | | 0.01 | | -0.04 | | 0.02 | 4166.67 |

**Group differences in summary measures of effort costliness (AUC, proportion of choices for the higher load/reward option) from the pre-scan effort discounting session remain non-significant when covarying for IQ and are unrelated to SES.**

Given the significant group difference in IQ (Table 1), we also conducted group comparisons of summary measures of effort costliness (AUC, proportion of choices for the higher load/reward option) accounting for IQ (one-way ANCOVAs). Results indicated no significant effect of IQ on either measure (both *F* <.5; both *p* > .5) and the group factor remained non-significant (both *F* <.7; both *p* > .4). Because valuation of monetary reward may be dependent on SES, we also explored relationships with summary measures of effort costliness. Mean SES in acAN (3.6 ± .11 SEM) did not differ from that in HC (3.9 ± .12 SEM; *t*_84_ = -1.7, *p* = .10) and no correlations with either AUC or the proportion of choices for the higher load/reward option were found either in the entire sample or in each group individually (all *r* < .17; all *p* > .24).


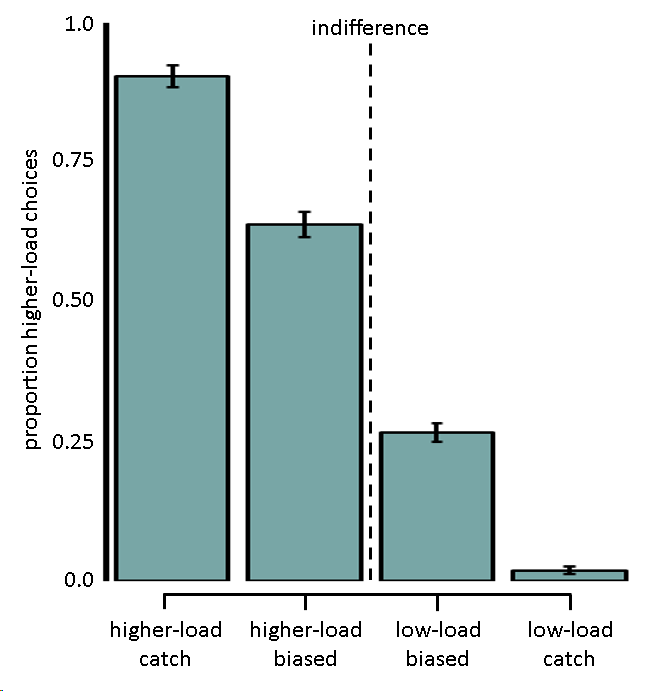


Fig. S3. Mean proportion of higher-load choices (± SEM) in all participants (*n* = 96) during the fMRI session (COGED Part 3) are plotted as a function of offer biasing and N-back load level. As in the COGED fMRI study of Westbrook et al. [3], the manipulation of effort-reward offers in the fMRI session based on the individual indifference points estimated during the pre-scan effort discounting procedure (COGED Part 2; described in detail above) reliably influenced the probability of choices for the higher-load (*N* = 2, 3 or 4) option (*F*_1,95_ = 1557.5; *p* < .001). Even after excluding “catch” trials, participants reliably chose higher-load offers on higher-load biased trials (choice probability > 0.5; *t*_95_ = 6.2; *p* < .001) and low-load offers on low-load biased trials (choice probability < 0.5; *t*_95_ = 13.6; *p* < .001).

**Correlation of individual summary measures of effort discounting between the pre-scan calibration and fMRI sessions**

Summary measures of effort discounting (AUC connecting SVs across *N*-back levels, proportion of choices for the higher load/reward option) from the pre-scan calibration and fMRI sessions were correlated in the entire sample (both *r* > .48; both *p* < .001) and within each group (all *r* > .46; all *p* < .001). No group differences were detected in either measure in either session (all *t* < .61; all *p* > .27).

**fMRI region of interest (ROI) analysis**

To further elucidate activation in a) the regions of the right LPFC and left IPL identified by the main whole-brain analyses of group differences in the parametric effect of chosen SV (Fig. 3) and SV_chosen_ - SV_unchosen_ (Fig. 4), respectively, and b) *a priori* defined ROIs (vmPFC, VS) derived from the literature [3, 13, 14], we extracted *β* parameter estimates corresponding to these SV regressors using MarsBaR toolbox for SPM (http://marsbar.sourceforge.net/) for each participant from all voxels located within a 6 mm radius sphere centered on peak coordinates and submitted the data to further analysis (independent samples *t*-tests and Pearson correlation analyses) in IBM SPSS (version 29) software, followed by multiple comparisons correction [15]. The coordinates of the vmPFC (2,46,-8) and VS (left: -12,12,-6; right: 12,10,-6) were taken from the aforementioned studies. Correlation analyses explored relationships between *β* estimates and relevant external variables in individuals with AN (EDI-2 total score, BMI-SMS, NFC).


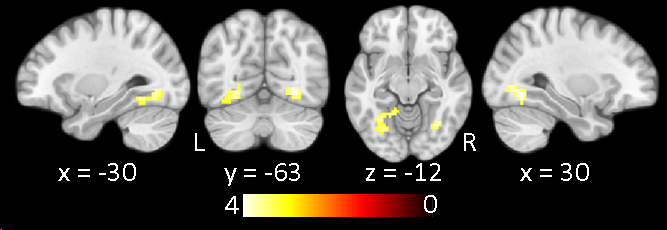


Fig. S4. **Main effect of activation associated with subjective value representation during the valuation phase.** Regions showing greater activation during the valuation phase of COGED part 3 as a function of trialwise SV of the first high-load/reward offer in the entire sample (*n* = 96) as revealed by parametric analysis is shown on selected slices of the MNI152 template brain at a voxelwise threshold of *p* < .001 (whole-brain corrected, *p* < .05). L = left; R = right. All significant correlations are summarized in Table S2.

| **Table S3** COGED Part 3 whole-brain results: Valuation phase activation correlated with first offer SV in the entire sample (*n* = 96) | | | | | | |
| --- | --- | --- | --- | --- | --- | --- |
|  | **Hemisphere** | **Voxels** | **X** | **y** | **z** | **t_max_** |
| fusiform/lingual gyrus | R | 79 | 30 | -63 | -8 | 4.49 |
| fusiform/lingual gyrus | L | 84 | -33 | -57 | -12 | 4.17 |
| cuneus | L/R | 41 | 3 | -93 | 19 | 4.08 |
| Note: Clusters are family-wise error rate corrected (*p* < .05) surviving a voxelwise cluster-forming threshold of *p* < .001. | | | | | | |


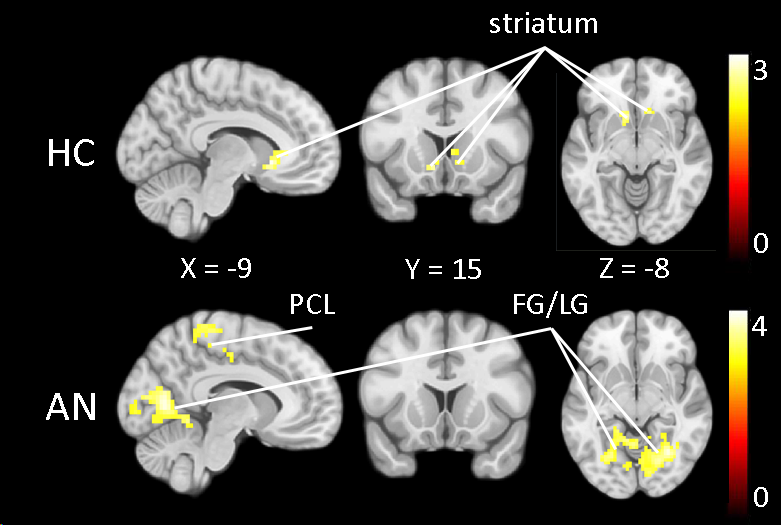


Fig. S5. **Activation associated with subjective value representation during the valuation phase in each group.** Regions showing greater activation during the valuation phase of COGED part 3 as a function of the trialwise SV of the first high-load/reward offer as revealed by parametric analysis is shown separately in the healthy control (HC) sample (*n* = 48; top row) and the anorexia nervosa (AN) sample (*n* = 48; bottom row) on selected slices of the MNI152 template brain at an exploratory voxelwise threshold of *p* < .01 (whole-brain corrected, *p* < .05). Consistent with previous fMRI studies of effort discounting [14] including most notably the study from which the current task was derived [3], the only region showing a relationship in the HC group was the striatum including ventral striatum (VS). In contrast, the AN group showed activation correlated with first offer SV in the same regions of visual cortex observed in analysis of the data from the entire sample (Fig. S4) including the regions of fusiform gyrus (FG) and lingual gyrus (LG), bilateral medial motor regions spanning from the supplementary motor area to the paracentral lobule (PCL) and regions of left frontoparietal cortex including the intraparietal sulcus and frontal eye field (neither visible in the shown slices). All correlations are summarized in Table S3.

| **Table S4** COGED Part 3 whole-brain results: Valuation phase activation correlated with first offer SV separately in the HC sample (*n* = 48) and the AN sample (*n* = 48) | | | | | | | | | | |
| --- | --- | --- | --- | --- | --- | --- | --- | --- | --- | --- |
|  | **Group** | **Anatomical Area** | **Hemisphere** | | | **Voxels** | **x** | **y** | **z** | **t_max_** |
|  | **HC** |  |  | | |  |  |  |  |  |
|  |  | striatum | | L/R | | 89 | 9 | 21 | 4 | 3.11 |
|  | **AN** |  | | | | | | | | |
|  |  | cuneus/fusiform/lingual/  middle occipital | | | L/R | 1265 | 3 | -93 | 15 | 4.42 |
|  |  | paracentral lobule/  supplementary motor area | | | L/R | 225 | 9 | -33 | 65 | 3.43 |
|  |  | middle frontal gyrus | | | L | 107 | -27 | 0 | 46 | 3.39 |
|  |  | intraparietal sulcus | | | L | 95 | -24 | -66 | 42 | 3.13 |
| Note: Clusters are family-wise error rate corrected (*p*_FWE_ < .05) surviving a voxelwise cluster-forming threshold of *p* < .01. See also Figure S5. | | | | | | | | | | |


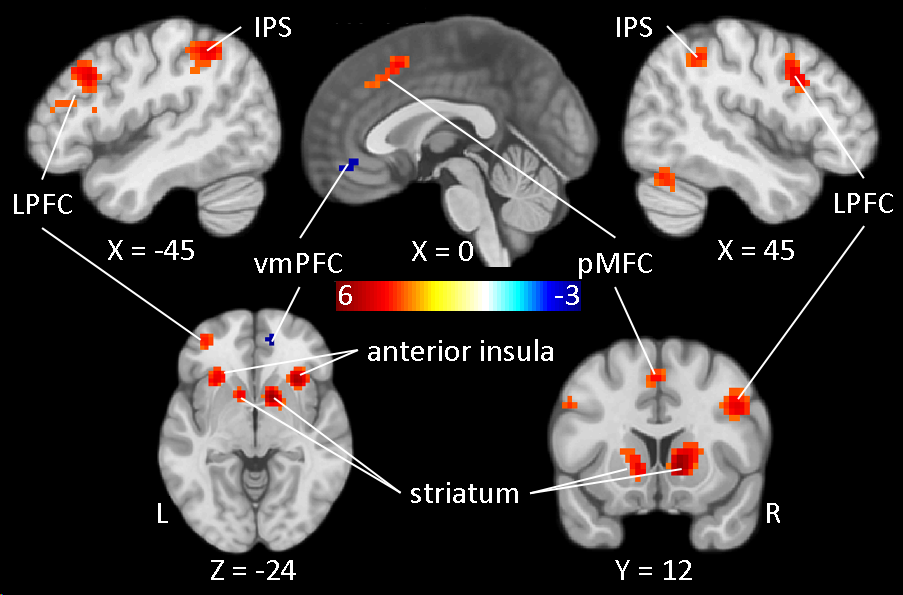


Figure S6. **Main effect of activation associated with subjective value representation of the chosen offer during decision-making.** Regions showing greater activation during the decision-making phase of COGED part 3 as a function of trialwise SV of the chosen offer in the entire sample (*n* = 96) as revealed by parametric analysis is shown on selected slices of the MNI152 template brain at a voxelwise threshold of *p* < .001 (whole-brain corrected, *p* < .05). Warm colors indicate positive correlations, while cool colors indicate negative relationships. LPFC = lateral prefrontal cortex; IPS = intraparietal sulcus; vmPFC = ventromedial prefrontal cortex; pMFC = posterior medial frontal cortex. L = left; R = right. All significant correlations are summarized in Table S4. Note that in addition to the negative correlation in vmPFC, a negative correlation was also present in posterior cingulate cortex at a more lenient exploratory voxelwise threshold of *p* < .01 (whole-brain corrected, *p* < .05); regions commonly ascribed to the brain’s default mode or “task-negative” network when co-activated [16], suggesting that the less participants valued the chosen option, the more they disengaged from decision-making and vice versa.

| **Table S5 COGED Part 3 whole-brain results: Decision phase activation correlated with chosen SV in the entire sample (*n*=96)** | | | | | | | |
| --- | --- | --- | --- | --- | --- | --- | --- |
| **Anatomical Area** | | **Hemisphere** | **Voxels** | **x** | **Y** | **z** | **t_max_** |
| ***positive correlation*** | | | | | | | |
| middle frontal gyrus | | L | 258 | -45 | 21 | 35 | 4.50 |
| precentral sulcus (frontal eye field) | | L | 35 | -30 | 0 | 38 | 4.54 |
| middle frontal gyrus | | R | 79 | 45 | 12 | 35 | 4.42 |
| dorsal anterior cingulate/pre-supplementary motor area | | L/R | 94 | -9 | 27 | 38 | 4.45 |
| striatum | | L | 155 | -21 | 3 | 15 | 4.43 |
| striatum | | R | 181 | 12 | 9 | 2 | 6.09 |
| intraparietal sulcus | | L | 341 | -30 | -60 | 35 | 5.92 |
| intraparietal sulcus | | R | 150 | 36 | -60 | 46 | 4.97 |
| cerebellum | |  | 121 | 36 | -60 | -27 | 4.24 |
| ***negative correlation*** | | | | | | | |
| medial frontal gyrus | L/R | | 50 | 12 | 57 | 4 | -3.68 |
| Note: Clusters are family-wise error rate corrected (*p*_FWE_ < .05) surviving a voxelwise cluster-forming threshold of *p* < .001. See also Figure S6. | | | | | | | |


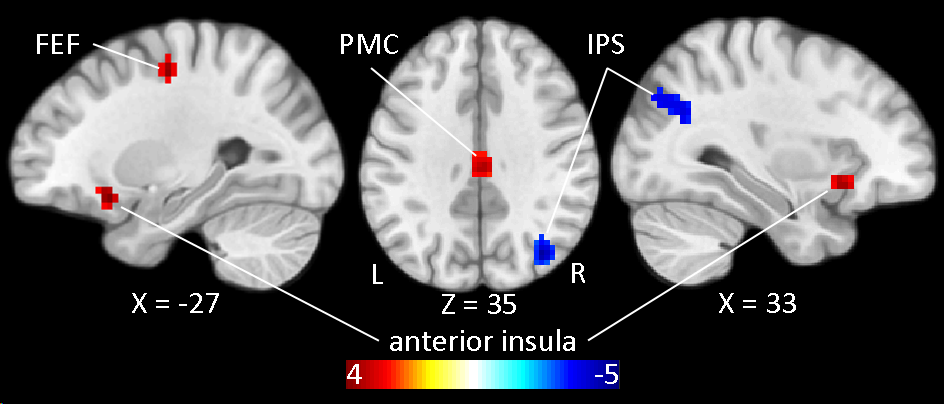


Figure S7. Main effect of **activation related to the decision easiness/difficulty.** Regions showing greater activation during the decision-making phase of COGED part 3 as a function of trialwise difference in SV between the chosen and unchosen options (SV_chosen_ - SV_unchosen_) in the entire sample (*n* = 96) as revealed by parametric analysis is shown on selected slices of the MNI152 template brain at a voxelwise threshold of *p* < .001 (whole-brain corrected, *p* < .05). Warm colors indicate positive correlations, while cool colors indicate negative relationships. FEF = frontal eye field; IPS = intraparietal sulcus; PMC = posterior midcingulate cortex; L = left; R = right. All significant correlations are summarized in Table S5.

| **Table S6 COGED Part 3 whole-brain results: Decision phase activation correlated with the difference between chosen SV - unchosen SV in the entire sample (n=96)** | | | | | | | |
| --- | --- | --- | --- | --- | --- | --- | --- |
| **Anatomical Area** | **Hemisphere** | | **Voxels** | **x** | **y** | **z** | **t_max_** |
| ***positive correlation*** | | | | | | | |
| anterior insula | | L | 32 | -30 | 18 | -15 | 4.87 |
| anterior insula | | R | 25 | 30 | 18 | -12 | 4.89 |
| mid-cingulate/posterior cingulate | | L/R | 44 | 3 | -24 | 35 | 4.20 |
| middle frontal gyrus (frontal eye field) | | L | 32 | -27 | -12 | 50 | 4.56 |
| ***negative correlation*** | | | | | | | |
| intraparietal sulcus | R | | 84 | 36 | -72 | 30 | -5.12 |
| Note: Clusters are family-wise error rate corrected (*p*_FWE_ < .05) surviving a voxelwise cluster-forming threshold of *p* < .001. See also Figure S7. | | | | | | | |


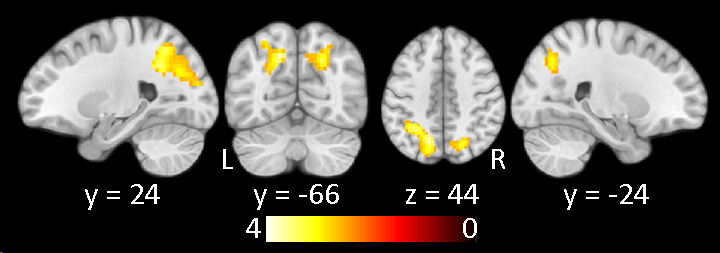


Figure S8. G**roup differences in** **activation related to the decision easiness/difficulty (exploratory).**  Regions showing greater activation during the decision-making phase of COGED part 3 in AN relative to HC (AN > HC) as a function of trialwise difference in SV between the chosen and unchosen options (SV_chosen_ - SV_unchosen_) as revealed by parametric analysis is shown on selected slices of the MNI152 template brain at an exploratory voxelwise threshold of *p* < .01 (whole-brain corrected, *p* < .05) to illustrate the bilateral nature of the relationship reported in the main article (Fig. 4). No other correlations emerged at this threshold.

**Group differences in SV-related fMRI activation in frontoparietal control regions remain significant when covarying for IQ.**

Given the significant group difference in IQ (Table 1), we tested whether the group differences in SV-related activation in the identified regions of right LPFC (Fig. 3) and left IPS (Fig. 4) remained when accounting for potential IQ effects by submitting the respective corresponding beta estimates to ANCOVAs. Results indicated no significant effect of IQ on SV-related activation in either region (both *F* <.9; both *p* > .4) and the group factor remained significant (both *F* > 9.2; both *p* < .003).

**Group differences in SV-related fMRI activation in valuation-related brain regions remain non-significant when covarying for IQ.**

Given the significant group difference in IQ (Table 1), we tested whether the lack of group differences in SV-related activation in the *a priori*-defined valuation-related ROIs (vmPFC, VS) remained when accounting for potential IQ effects by submitting the respective corresponding beta estimates from both the valuation and decision-making phases to ANCOVAs. Results indicated no significant effect of IQ on SV-related activation in either region during either task phase (all *F* < 2.5; all *p* > .1) and the group factor remained non-significant (all *F* < 2.8; all *p* > .1).

**Bayesian analysis of SV-related fMRI activation in valuation-related brain regions underline the null hypothesis of no group differences.**

One of our main hypotheses was that SV-related activation in reward-related ROIs (vmPFC, VS) during either the valuation or decision-making phases of COGED would differ between AN and HC. Contrary to this hypothesis, however, the results obtained with both whole brain analysis and traditional null hypothesis significance testing of *β* parameter estimates extracted from these regions indicated no clear group differences. To provide more evidence in support of the null hypothesis of no group differences in these ROIs, we conducted Bayesian ANCOVAs (again covarying for IQ because of the group difference; see previous section of the Supplementary Information) with JASP software (https://jasp-stats.org/) using the respective activation parameters extracted from these regions. Here the evidence in all 6 cases [3 ROIs (vmPFC, VS_right_, VS_left_) during the 2 COGED phases of interest (valuation and decision-making)] ranged from weak (BF_01_ = 2.5 in VS_left_ during decision-making) to strong (BF_01_ = 10.6 in vmPFC during valuation) favoring the null hypothesis of no group difference.

References

1. Munkres J. Algorithms for the assignment and transportation problems. J Soc Ind Appl Math. 1957;5:32–38.

2. Ganzeboom HBG, De Graaf PM, Treiman DJ. A standard international socio-economic index of occupational status. Soc Sci Res. 1992;21:1–56.

3. Westbrook A, Lamichhane B, Braver T. The Subjective Value of Cognitive Effort is Encoded by a Domain-General Valuation Network. J Neurosci Off J Soc Neurosci. 2019;39:3934–3947.

4. Bernardoni F, King JA, Geisler D, Ritschel F, Schwoebel S, Reiter AMF, et al. More by stick than by carrot: A reinforcement learning style rooted in the medial frontal cortex in anorexia nervosa. J Abnorm Psychol. 2021;130:736–747.

5. Culbreth A, Westbrook A, Barch D. Negative symptoms are associated with an increased subjective cost of cognitive effort. J Abnorm Psychol. 2016;125:528–536.

6. Westbrook A, Kester D, Braver TS. What is the subjective cost of cognitive effort? Load, trait, and aging effects revealed by economic preference. PloS One. 2013;8:e68210.

7. Hart SG, Staveland LE. Development of NASA-TLX (Task Load Index): Results of Empirical and Theoretical Research. Adv. Psychol., vol. 52, Elsevier; 1988. p. 139–183.

8. Chang WC, Westbrook A, Strauss GP, Chu AOK, Chong CSY, Siu CMW, et al. Abnormal cognitive effort allocation and its association with amotivation in first-episode psychosis. Psychol Med. 2020;50:2599–2609.

9. R Core Team. R: A language and environment for statistical computing. 2022.

10. Bates D, Mächler M, Bolker B, Walker S. Fitting Linear Mixed-Effects Models Using lme4. J Stat Softw. 2015;67.

11. Bürkner P-C. Advanced Bayesian Multilevel Modeling with the R Package brms. R J. 2018;10:395.

12. Aschenbrenner AJ, Crawford JL, Peelle JE, Fagan AM, Benzinger TLS, Morris JC, et al. Increased cognitive effort costs in healthy aging and preclinical Alzheimer’s disease. Psychol Aging. 2023;38:428–442.

13. Bartra O, McGuire JT, Kable JW. The valuation system: a coordinate-based meta-analysis of BOLD fMRI experiments examining neural correlates of subjective value. NeuroImage. 2013;76:412–427.

14. Lopez-Gamundi P, Yao Y-W, Chong TT-J, Heekeren HR, Mas-Herrero E, Marco-Pallarés J. The neural basis of effort valuation: A meta-analysis of functional magnetic resonance imaging studies. Neurosci Biobehav Rev. 2021;131:1275–1287.

15. Benjamini Y, Hochberg Y. Controlling the False Discovery Rate: A Practical and Powerful Approach to Multiple Testing. J R Stat Soc Ser B Methodol. 1995;57:289–300.

16. Uddin LQ, Kelly AM, Biswal BB, Castellanos FX, Milham MP. Functional connectivity of default mode network components: correlation, anticorrelation, and causality. Hum Brain Mapp. 2009;30:625–637.
